# Reference intervals for urine creatinine-to-specific gravity ratio as an indicator of relative creatinine excretion rate: a cross-sectional study

**DOI:** 10.1097/MS9.0000000000003511

**Published:** 2025-06-23

**Authors:** Shih-Ping Hsu, Chiang-Ting Chien

**Affiliations:** a Department of Internal Medicine, Far Eastern Memorial Hospital, New Taipei, Taiwan; b School of Life Science, National Taiwan Normal University, Taipei, Taiwan; c College of Medicine, National Taiwan University, Taipei, Taiwan; d General Education Center, Lunghwa University of Science and Technology, Taoyuan, Taiwan

**Keywords:** acute kidney injury, biomarker, National Health And Nutrition Examination Survey, urine creatinine-to-osmolality ratio, urine specific gravity

## Abstract

**Background:**

This study aimed to establish reference intervals for the spot urine creatinine-to-specific gravity difference (SGD; the last two digits of specific gravity) ratio (sUCr/SGD) as an alternative to the spot urine creatinine-to-osmolality ratio (sUCr/Osm) for indicating urinary creatinine excretion rate.

**Methods:**

Data from 3288 adults aged 18–79.9 years without overt proteinuria or glucosuria from the NHANES 2007–2008 survey were analyzed. Parameters including age, sex, blood urea nitrogen (BUN), and serum creatinine were obtained. Spot urine creatinine and specific gravity values were measured and subjected to multivariable regression analysis to predict the estimated sUCr/SGD (esUCr/SGD) for each individual.

**Results:**

The mean age of participants was 47 ± 17 years, with 54.0% being male. The mean BUN was 12.6 ± 4.6 mg/dL, and the mean serum creatinine was 0.86 ± 0.22 mg/dL. The mean values for spot urine creatinine and SGD were 123.6 ± 75.4 mg/dL and 16.8 ± 6.9, respectively, resulting in an sUCr/SGD of 7.0 ± 2.4. A formula for esUCr/SGD was developed considering interpersonal variations. Less than 5% of individuals exhibited sUCr/SGD values below 3.6 or an index to esUCr/SGD below 0.60.

**Conclusions:**

The study concluded that with a left-sided reference limit of <3.6 for absolute values or <0.60 for relative index, sUCr/SGD may serve as an acceptable alternative to sUCr/Osm in estimating the relative urinary excretion rate of creatinine.

## Introduction

The renal creatinine (Cr) clearance rate, derived from the urinary Cr excretion rate divided by the concurrent serum Cr (SCr) concentration, remains a widely accepted surrogate indicator of renal excretory function in experimental research and clinical practice^[1,2]^. It is plausible to hypothesize that when the urinary Cr excretion rate surpasses a certain threshold, it may indicate a reduced likelihood of acute kidney injury, even among patients having preexisting chronic kidney disease with elevated but stable SCr levels for over 3 months[3]. Consequently, developing a simple method to detect acute changes in urinary Cr excretion could enable real-time monitoring of kidney function.

A spot urine Cr-to-osmolality ratio (sUCr/Osm) has been proposed to estimate urinary Cr excretion rate[1] and to help rule out acute kidney disease in outpatient settings[4]. In typical adults, this ratio averages about 1.9 ± 0.8 mg of Cr per milliosmole (Cr/mOsm), with a left-sided, lower reference limit of approximately 0.8 mg Cr/mOsm[3]. The physiological basis is that human kidneys excrete predictable amounts of waste products (including Cr) and osmolar load^[5–8]^ in a steady and parallel manner[9] to maintain the homeostasis. A reduced sUCr/Osm may indicate inadequate Cr excretion and subsequent accumulation in the body.

While routine laboratory tests measure spot urine Cr (sUCr) and osmolality (UOsm), urine specific gravity (USG) is often used as a more economical alternative to UOsm. USG correlates well with UOsm^[10–12]^ and typically increases by approximately 0.001 for every 35–40 mOsm/kg rise in urine osmolality (UOsm)^[13,14]^. For clarity, the last two digits of USG, representing 1000-fold the difference in specific gravities between urine and pure water, are referred to as specific gravity difference (SGD).

Building on the established prognostic value of sUCr/Osm[4], it is logical to explore SGD as a substitute for osmolality. This study aims to evaluate the suitability of the spot urine Cr-to-SGD ratio (sUCr/SGD) as an alternative method for estimating urinary Cr excretion in the general adult population. By examining the distribution and reference intervals of sUCr/SGD and comparing them with those of sUCr/Osm, our findings could lay the groundwork for future clinical validation trials.

## Materials and methods

### Study population

This cross-sectional study utilized data from the National Health and Nutrition Examination Survey (NHANES). Only in NHANES 2007–2008 were urinary Cr and USG measured simultaneously, while urinary Cr was measured with concurrent UOsm in NHANES 2009–2010 and 2011–2012. In NHANES 2007–2008, 12 943 individuals were screened, with 10 149 completing the interview and 9762 examined. Data from 9762 individuals were first released in September 2009[15].

**Figure 1.**
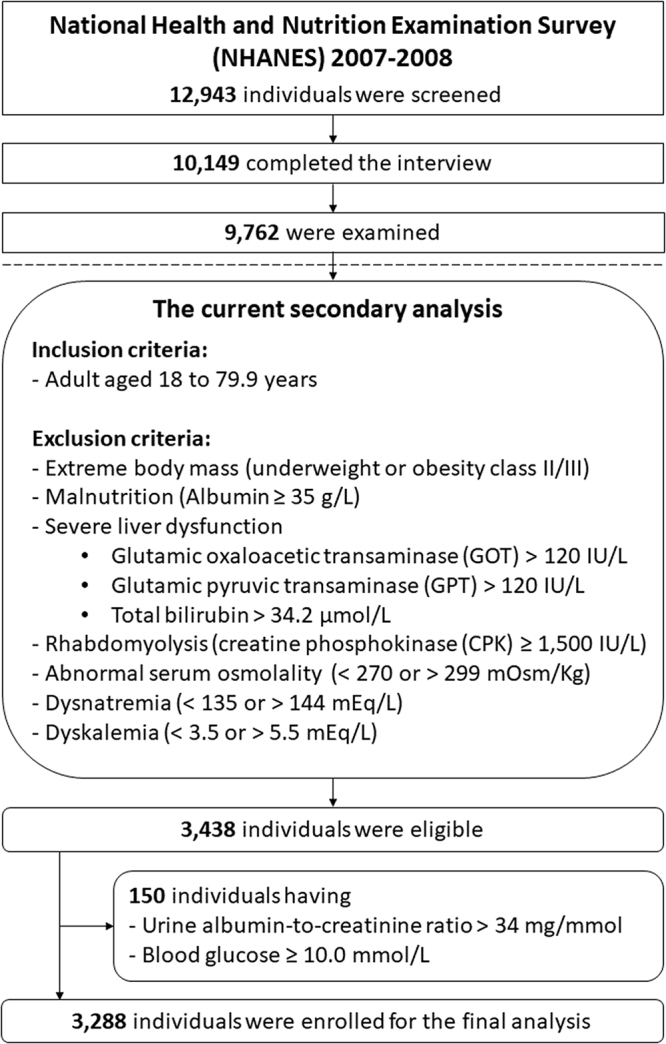
The sampling flowchart.

For the sake of comparison, the methodology utilized for participant screening is derived from that previously described in the published work[3]. In brief, participants aged 18–79.9 years were included. Individuals aged 80 years and older were excluded due to top-coding. Following the initial exclusion based on anthropometric and laboratory parameters to exclude known conditions associated with extraordinary urinary Cr and osmoles excretion, 3438 participants were deemed eligible. In consideration of conditions that result in an overestimation of UOsm inferred by SGD, an additional 150 subjects exhibiting urine albumin-to-Cr ratios exceeding 300 mg/g or blood glucose levels of 180 mg/dL were further excluded. The final dataset comprised 3288 participants. The sampling process is illustrated in Figure 1.

The NHANES 2007–2008 study received approval from the institutional review board at the National Center for Health Statistics. All participants provided oral and written informed consent. According to the U.S. Federal Policy for the Protection of Human Subjects (45 CFR 46), the current secondary analysis study using publicly available, existing, de-identified data, such as NHANES 2007–2008, is not considered research involving human subjects and therefore does not require review by an institutional review board (IRB) or a Committee for Protection of Human Subjects (CPHS). This analysis used fully de-identified, publicly available data with no linkage to individual participants. This secondary analysis study was conducted in accordance with the Declaration of Helsinki. All procedures for processing data and interpreting results were conducted in adherence to the guidelines prescribed therein. This work has been reported in line with the STROCSS criteria[16].

### Demographics, comorbidities, and medication use

Demographic information (age, sex, race) was gathered during interviews.

Hypertension was defined by physician diagnosis, antihypertensive medication use, or blood pressure criteria. Diabetes mellitus was defined by physician diagnosis, diabetic medication use, or blood glucose levels. Concurrent medical conditions were determined via self-reported data. Prescription medication use was categorized into angiotensin-converting enzyme inhibitors (ACEIs), angiotensin II receptor blockers (ARBs), β-adrenergic blocker, calcium channel blocker, diuretics, and other types.

### Measurement of blood biochemistry, urine creatinine, and USG

Blood and urine samples were collected at mobile examination centers.

Serum samples were analyzed by Collaborative Laboratory Services. The NHANES Laboratory/Medical Technologists Procedures Manual provided instructions for sample collection and processing. The vials were kept frozen at −30°C until their shipment to the National Center for Environmental Health for testing. SCr was measured using the Jaffé rate method (kinetic alkaline picrate) on the Beckman Synchron LX20 in 2007 and the Beckman Coulter UniCel® DxC800 in 2008, with isotope dilution mass spectrometry standardization.

**HIGHLIGHTS**

- Objective and background: The study aims to establish reference intervals for the spot urine creatinine-to-specific gravity difference (SGD) ratio (sUCr/SGD) as an alternative to the spot urine creatinine-to-osmolality ratio (sUCr/Osm) for indicating urinary creatinine excretion rate.
- Methods: Data from 3288 adults aged 18–79.9 years without overt proteinuria or glucosuria from the NHANES 2007–2008 survey were analyzed. Parameters including age, sex, blood urea nitrogen (BUN), and serum creatinine were obtained. Spot urine creatinine and specific gravity values were measured and subjected to multivariable regression analysis to predict the estimated sUCr/SGD (esUCr/SGD) for each individual.
- Results: The mean age of participants was 47 ± 17 years, with 54.0% being male. The mean BUN was 12.6 ± 4.6 mg/dL, and the mean serum creatinine was 0.86 ± 0.22 mg/dL. The mean values for spot urine creatinine and SGD were 123.6 ± 75.4 mg/dL and 16.8 ± 6.9, respectively, resulting in an sUCr/SGD of 7.0 ± 2.4. A formula for esUCr/SGD was developed considering interpersonal variations.
- Conclusions: The study concluded that with a left-sided reference limit of <3.6 for absolute values or <0.60 for relative index, sUCr/SGD may serve as an acceptable alternative to sUCr/Osm in estimating the relative urinary excretion rate of creatinine.
- Clinical implications: The reference intervals for sUCr/SGD are comparable to those of sUCr/Osm, making sUCr/SGD a practical alternative in clinical settings to monitor kidney function trends.

Urine samples were processed and shipped to a central laboratory. For urinary Cr analysis, urine samples, either spot or timed, were stored at 2°C–8°C until analysis within 36 hours of receipt at the laboratory. In 2007–2008, urinary Cr was measured using either an enzymatic method (creatinase) on a Roche/Hitachi Modular P Chemistry Analyzer or the Jaffé rate method (kinetic alkaline picrate) on a Beckman Synchron CX3 Clinical Analyzer. For USG, approximately 0.3 mL of a well-mixed urine sample was placed on the prism surface and measured using the digital hand-held refractometer ATAGO PAL-10S.

### Statistical analysis

NHANES 2007–2008 data were accessed through SAS transport files[15]. The survey respondents were identified with a unique sequence number used to merge the data files. For the current secondary analysis, IBM® SPSS® Statistics 22.0 (New York, USA) was used to process, filter, and analyze the data.

Continuous variables are presented as mean ± standard deviation (SD), while categorical variables are shown as number (%). To examine the relationship between urine profile (sUCr, SGD, and sUCr/SGD) and factors such as demographics, comorbidities, medication use, and laboratory data, differences in binary/categorical variables were first assessed using Student’s t-test, chi-squared test, or one-way analysis of variance with Bonferroni post hoc test. Correlations with continuous variables were assessed using univariable regression. The logarithmic models were found to have better fitness, and thus, all variables were logarithmically transformed before being analyzed with linear regression models, following the established methodology[3]. Multivariable regression was then employed to further explore the relationships between urine profiles and the biologically plausible and statistically significant variables. The basic multivariable regression model included four primary factors related to endogenous Cr generation – age, sex, body weight (BW), and race^[5,6]^ – as fixed covariables. The effect sizes of other statistically significant variables were evaluated using the stepwise method. Two-tailed tests with a *P*-level <0.05 were considered statistically significant.

## Results

### Demographics, comorbidities, and medication use

Of the 3288 adults who met the criteria, the mean age was 47 ± 17 years, with 46.0% being female. The mean BW was 76.1 ± 14.6 kg. A total of 17.9% identified as Black. The prevalence of hypertension was 36.2%, and 9.7% had diabetes mellitus. Among them, 12.8% were taking ACEIs or ARBs, and 9.1% were on diuretics (Table 1).

**Table 1:** Demographics, comorbidities, medication use, and laboratory data (*n* = 3288)

	Mean ± SD [IQR] or number (%)
Age, year	46.5 ± 17.2 [32.0–61.0]
Female sex	1512 (46.0)
Body weight, kg	76.4 ± 14.6 [65.6–86.2]
Body mass index, kg/m^2^	26.9 ± 4.0 [23.8–30.0]
Race	
Mexican American	606 (18.4)
Other Hispanic	405 (12.3)
Non-Hispanic White	1527 (46.4)
Non-Hispanic Black	587 (17.9)
Other Races	163 (5.0)
Comorbidities	
Hypertension	1190 (36.2)
Diabetes mellitus	319 (9.7)
Coronary artery disease	165 (5.0)
Congestive heart failure	55 (1.7)
Cerebrovascular disease	83 (2.5)
Active liver disease	48 (1.5)
Cancer	249 (7.6)
Medication use	
ACEI/ARB	422 (12.8)
β-adrenergic blocker	271 (8.2)
Calcium channel blocker	153 (4.7)
Diuretics	300 (9.1)
Other antihypertensives	91 (2.8)
Blood biochemistry	
Urea Nitrogen, mg/dL	12.6 ± 4.6 [9–15]
Creatinine, mg/dL	0.86 ± 0.22 [0.72–1.00]
MDRD eGFR, mL/min/1.73 m^2^	93.7 ± 24.4 [77.3–107.5]
Albumin, g/dL	4.3 ± 0.33 [4.1–4.5]
GOT, IU/L	25.2 ± 8.5 [20–28]
GPT, IU/L	24.7 ± 12.8 [17–28]
Total bilirubin, mg/dL	0.8 ± 0.3 [0.6–0.9]
Glucose, mg/dL	93.6 ± 17.2 [85–101]
Osmolality, mOsm/Kg	278 ± 4 [275–281]
Na, meq/L	139.5 ± 1.8 [138–141]
K, meq/L	4.0 ± 0.3 [3.8−4.2]
Urine profile	
Creatinine, mg/dL	123.6 ± 75.4 [65–168]
Specific gravity	1.017 ± 0.007 (1.002–1.038)
SGD	16.8 ± 6.9 [11.0–22.0]
sUCr/SGD	7.0 ± 2.4 [5.3–8.3]
UACR, mg/g	14 ± 26 [4–12]

ACEI, angiotensin-converting enzyme inhibitor; ARB, angiotensin II receptor blocker; CI, confidence interval; creatinine to μmol/L, 88.4; glucose to mmol/L, 0.0555; GOT, glutamic oxaloacetic transaminase; GPT, glutamic pyruvic transaminase; IQR, interquartile range; MDRD eGFR, estimated glomerular filtration rate derived with Modification of Diet in Renal Disease (MDRD) Study equation; SD, standard deviation; SGD, specific gravity difference (a value that is 1000-fold the difference in specific gravities between urine and pure water); sUCr/SGD, spot urine creatinine-to-SGD ratio; total bilirubin to μmol/L, 17.1; UACR to mg/mmol, 0.113; UACR, urine albumin to creatinine ratio to convert albumin to g/L, multiplied by 10; and urea nitrogen to mmol/L, 0.357.

### Blood biochemistry and urine profile

The blood urea nitrogen (BUN) level was 12.6 ± 4.6 mg/dL and the SCr level was 0.86 ± 0.22 mg/dL. Spot urine samples demonstrated an sUCr level of 124 ± 75 mg/dL, a USG of 1.017 ± 0.007, an SGD of 16.8 ± 6.9, and an sUCr/SGD ratio was 7.0 ± 2.4 with a median of 6.7 (Table 1).

### Factors correlating with urine profile

The differences regarding binary/categorical variables between the urine profile (sUCr, SGD, and sUCr/SGD, respectively) and demographics, comorbidities, medication use, and laboratory data are present in Table 2. Correlations of continuous variables using univariable regression are displayed in Table 3. Notably, BW was chosen as a proxy for body size over body mass index due to its larger effect size, as indicated by the standardized coefficients (0.255 vs. 0.069). The results of the standardized (β) coefficients of significant covariables in the multiple regression models are demonstrated in Table 4.

**Table 2:** Comparisons of spot urine creatinine, specific gravity difference^a^, and creatinine-to-specific gravity difference ratio, in terms conditions of demographics, comorbidities, and medication use

	sUCr, mg/dL	*P*	Specific gravity difference	*P*	sUCr/SGD	*P*
	Mean ± SD		Mean ± SD		Mean ± SD	
Sex						
Male vs. female	142.3 ± 76.3 vs. 101.6 ± 68.2	<0.001	18.1 ± 6.6 vs. 15.2 ± 7.0	<0.001	7.57 ± 2.40 vs. 6.33 ± 2.23	<0.001
Race						
Mexican American	118.9 ± 70.2	<0.001	17.6 ± 6.9	<0.001	6.50 ± 2.17	<0.001
Other Hispanic	118.8 ± 68.9		17.2 ± 6.9		6.64 ± 2.12	
Non-Hispanic White	113.0 ± 70.4		15.7 ± 6.8		6.81 ± 2.25	
Non-Hispanic Black	162.4 ± 86.0		18.7 ± 6.6		8.39 ± 2.69	
Other races	113.2 ± 70.9		16.4 ± 6.8		6.52 ± 2.26	
Black race (yes vs. no)	162.4 ± 86.0 vs.115.2 ± 70.2	<0.001	18.7 ± 6.6 vs. 16.4 ± 6.9	<0.001	8.39 ± 2.69 vs. 6.70 ± 2.22	<0.001
Comorbidities (yes vs. no)						
Hypertension	113.9 ± 71.3 vs. 129.1 ± 77.2	<0.001	15.9 ± 6.5 vs. 17.3 ± 7.0	<0.001	6.79 ± 2.42 vs. 7.12 ± 2.38	<0.001
Diabetes mellitus	118.8 ± 70.1 vs. 124.1 ± 76.0	0.201	17.0 ± 6.4 vs. 16.8 ± 7.0	0.509	6.69 ± 2.46 vs. 7.03 ± 2.40	0.014
Coronary artery disease	111.3 ± 70.0 vs. 124.3 ± 75.7	0.032	15.8 ± 6.4 vs. 16.8 ± 6.9	0.076	6.59 ± 2.33 vs. 7.02 ± 2.40	0.025
Congestive heart failure	129.3 ± 85.7 vs. 123.5 ± 75.3	0.573	16.0 ± 6.3 vs. 16.8 ± 6.9	0.377	7.51 ± 3.07 vs. 6.99 ± 2.38	0.214
Cerebrovascular disease	108.9 ± 58.9 vs. 124.0 ± 75.8	0.025	15.8 ± 6.5 vs. 16.8 ± 6.9	0.194	6.73 ± 2.16 vs. 7.01 ± 2.40	0.297
Active liver disease	126.3 ± 64.1 vs. 123.6 ± 75.6	0.804	17.2 ± 5.8 vs. 16.8 ± 6.9	0.627	7.22 ± 2.31 vs. 7.00 ± 2.40	0.515
Cancer	107.9 ± 66.6 vs. 124.9 ± 76.0	<0.001	15.2 ± 6.5 vs. 16.9 ± 6.9	<0.001	6.73 ± 2.42 vs. 7.02 ± 2.39	0.067
Medication use (yes vs. no)						
ACEI/ARB	110.7 ± 66.8 vs. 125.5 ± 76.5	<0.001	15.9 ± 6.1 vs. 16.9 ± 7.0	0.001	6.65 ± 2.42 vs. 7.05 ± 2.39	0.001
β-adrenergic blocker	110.1 ± 71.9 vs. 124.8 ± 75.7	0.001	15.3 ± 6.0 vs. 16.9 ± 7.0	<0.001	6.81 ± 2.52 vs. 7.02 ± 2.39	0.183
Calcium channel blocker	111.0 ± 63.9 vs. 124.2 ± 75.9	0.015	15.7 ± 6.2 vs. 16.8 ± 6.9	0.030	6.77 ± 2.22 vs. 7.01 ± 2.40	0.234
Diuretics	106.7 ± 61.2 vs. 125.3 ± 76.5	<0.001	15.6 ± 5.9 vs. 16.9 ± 7.0	0.001	6.52 ± 2.27 vs. 7.05 ± 2.40	<0.001
Other antihypertensives	128.8 ± 79.4 vs. 123.5 ± 75.3	0.505	17.5 ± 6.9 vs. 16.8 ± 6.9	0.280	6.89 ± 2.20 vs. 7.00 ± 2.40	0.661

ACEI, angiotensin-converting enzyme inhibitor; ARB, angiotensin II receptor blocker; SGD, specific gravity difference (a value that is 1000-fold the difference in specific gravities between urine and pure water).

**Table 3:** Standardized (β) coefficients of univariable linear regression models for spot urine profile

	sUCr	*P*	SGD	*P*	sUCr/SGD	*P*
Age	−0.226	<0.001	−0.177	<0.001	−0.208	<0.001
Body weight	0.257	<0.001	0.190	<0.001	0.255	<0.001
Body mass index	0.114	<0.001	0.115	<0.001	0.069	<0.001
Blood biochemistry						
Urea nitrogen	0.055	0.002	0.197	<0.001	−0.174	<0.001
Creatinine	0.244	<0.001	0.103	<0.001	0.354	<0.001
MDRD eGFR	0.028	0.106	0.099	<0.001	−0.087	<0.001
Albumin	0.022	0.212	0.002	0.914	0.042	0.016
GOT	0.041	0.020	0.030	0.090	0.041	0.019
GPT	0.090	<0.001	0.092	<0.001	0.051	0.003
Total bilirubin	0.109	<0.001	0.052	0.003	0.148	<0.001
Glucose	0.013	0.458	0.040	0.022	−0.032	0.070
Osmolality	0.106	<0.001	0.160	<0.001	−0.014	0.407
Na	0.097	<0.001	0.090	<0.001	0.068	<0.001
K	−0.032	0.063	−0.011	0.546	−0.052	0.003
Urine profile						
sUCr	NA	NA	0.896	<0.001	0.761	<0.001
SGD	0.896	<0.001	NA	NA	0.393	<0.001
sUCr/SGD	0.761	<0.001	0.393	<0.001	NA	NA
UACR	−0.101	<0.001	−0.068	<0.001	−0.110	<0.001

GOT, glutamic oxaloacetic transaminase; GPT, glutamic pyruvic transaminase; MDRD eGFR, estimated glomerular filtration rate derived with Modification of Diet in Renal Disease (MDRD) Study equation; NA, non-applicable; sUCr, spot urine creatinine; SGD, specific gravity difference (a value that is 1000-fold the difference in specific gravities between urine and pure water); sUCr/SGD, spot urine creatinine-to-specific gravity difference ratio; UACR, urine albumin to creatinine ratio.

**Table 4:** Standardized (β) coefficients of multivariable linear regression models for spot urine profile

	sUCr	*P*	SGD	*P*	sUCr/SGD	*P*
Age	−0.043	<0.001	−0.008	0.302	−0.176	<0.001
Sex (ref. male)	0.028	0.001	−0.053	<0.001	−0.011	0.586
Body weight	0.042	<0.001	−0.016	0.042	0.122	<0.001
Black race (ref. non-Black race)	0.027	<0.001	0.003	0.687	0.135	<0.001
Comorbidities (ref. no presence)						
Hypertension	–a	–a	–a	–a	–a	–a
Diabetes mellitus					–a	–a
CAD	–a	–a			–a	–a
CVA	–a	–a				
Cancer	–a	–a	–a	–a		
Medication use (ref. no user)						
ACEI/ARB	–a	–a	–a	–a	–a	–a
β-adrenergic blocker	–a	–a	–a	–a		
CCB	–a	–a	–a	–a		
Diuretics	–a	–a	–a	–a	−0.036	0.022
Blood biochemistry						
Urea nitrogen	−0.191	<0.001	0.225	<0.001	−0.270	<0.001
Creatinine	0.218	<0.001	−0.227	<0.001	0.407	<0.001
Albumin					−0.042	0.008
GPT	–a	–a	–a	–a	–a	–a
Total bilirubin	0.038	<0.001	−0.037	<0.001	0.090	<0.001
Glucose			–a	–a		
Osmolality	–a	–a	0.017	0.024		
Na	–a	–a	–a	–a	0.056	<0.001
K					−0.084	<0.001
Urine profile						
sUCr	NA	NA	0.928	<0.001	NA	NA
SGD	0.896	<0.001	NA	NA	NA	NA
UACR	–a	–a	–a	–a	–a	–a
Adjusted R-squared	0.867	<0.001	0.862	<0.001	0.317	<0.001

CCB, calcium channel blocker; CVA, cerebrovascular accident; GOT, glutamic oxaloacetic transaminase; LDH, lactate dehydrogenase; NA, non-applicable; ref., reference group; sUCr, spot urine creatinine; SGD, specific gravity difference (a value that is 1000-fold the difference in specific gravities between urine and pure water); sUCr/SGD, spot urine creatinine-to-specific gravity difference ratio; UACR, urine albumin to creatinine ratio.

^a^ Omitted for clarity, as the *P*-value ≥0.05.

In its respective multivariable regression model with the four forced-in variables, sUCr demonstrated a negative correlation with age and BUN, as well as a positive correlation with female sex, BW, Black race, SCr, total bilirubin, and SGD. With regard to the model for SGD, SGD exhibited a negative correlation with female sex, BW, SCr, and total bilirubin, as well as a positive correlation with BUN, serum osmolality, and sUCr. The model for sUCr/SGD revealed that sUCr/SGD exhibited a negative correlation with age, diuretic use, BUN, serum albumin, and K, as well as a positive correlation with BW, Black race, SCr, total bilirubin, and Na.

### Distribution of spot urine creatinine-to-SGD ratio

**Figure 2.**
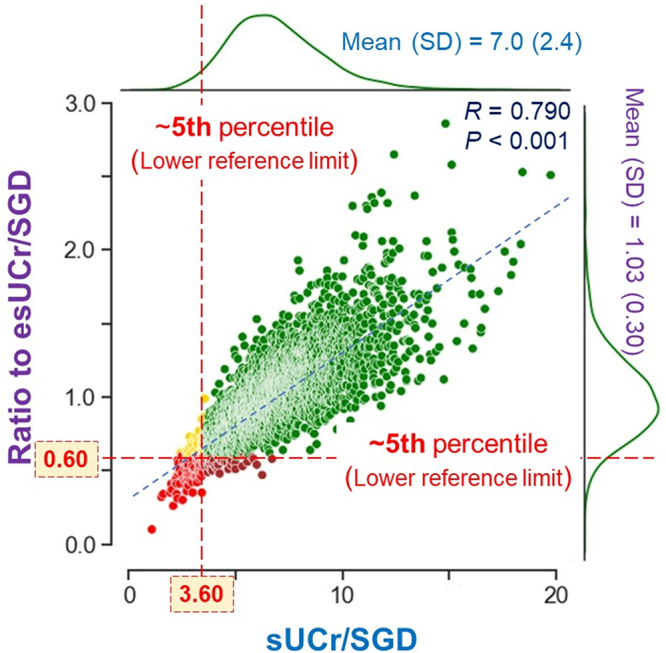
Respective distributions and their correlations regarding spot urine creatinine-to-specific gravity difference (SGD) ratio (sUCr/SGD) and the sUCr/SGD values indexed for personalized estimated sUCr/SGD (ratio to esUCr/SGD). The distribution patterns of these two parameters are delineated with green lines on the margins. The values of the 5th percentile are indicative of the lower reference limits of their respective left-sided 95% reference intervals. Green dots, above both 5th percentiles; yellow or brown dots, only below either 5th percentile; and red dots, below both 5th percentiles. SD, standard deviation.

The mean of the absolute value of sUCr/SGD is 7.0 ± 2.4, ranging 1.1–19.8, with a median of 6.7 (Table 1 and Fig. 2).

In order to gain a more nuanced understanding of the distribution of sUCr/SGD, in addition to the sUCr/SGD values derived directly from the laboratory data, it was of interest to consider the relative values of sUCr/SGD indexed to the personalized estimated values of sUCr/SGD (esUCr/SGD), incorporating relevant composite factors for a given individual.

To develop a practical and succinct formula for esUCr/SGD, only covariables with standardized coefficients greater than 0.10 and *P*-values less than 0.001 in Table 4 were selected as potential candidates. This approach aimed to achieve an optimal balance between the number of predictors and the gain in adjusted *R*-squared. As sex was excluded due to its lack of statistical significance, the selected variables were age, BW, Black race, BUN, and SCr. Subsequently, the esUCr/SGD for a given individual was calculated as 8.440 × (age, year)^−0.169^ × (BW, kg)^0.262^ × 1.164 [if Black race] × (BUN, mg/dL)^−0.263^ × (serum Cr, mg/dL)^0.591^, with the adjusted *R*-squared = 0.299. The mean value of esUCr/SGD was 6.8 ± 1.4, ranging 3.6–13.3, with a median of 6.6. The mean value of the sUCr/SGD indexed to esUCr/SGD was 1.03 ± 0.30, ranging 0.10–2.86, with a median of 1.00.

The distributions and correlation of the absolute and relative index values of sUCr/SGD are illustrated in Figure 2.

### Reference intervals of spot urine creatinine-to-SGD ratio

Unlike clinical decision limits, which are diagnostic thresholds employed to ascertain the presence of a particular disease or a markedly elevated risk of unfavorable clinical outcomes[17], reference intervals for tests such as sUCr/SGD, indicative of the relative urinary Cr excretion rate, are founded upon results observed in 95% of the reference population^[18,19]^. Given that only low renal excretion of Cr may be clinically relevant, it is pertinent to consider the lower one-sided reference limit of the 5th percentile and the left-sided 95% reference interval for sUCr/SGD. Further analysis revealed that 156 individuals (4.7%, below the 5th percentile) exhibited absolute sUCr/SGD values less than 3.60, while 152 individuals (4.6%) demonstrated relative sUCr/SGD values indexed to esUCr/SGD less than 0.60. In 104 participants (3.2%), the sUCr/SGD levels were below the lower reference limits of both the left-sided reference intervals stated above. Figure 2 provides a visual summary.

### Comparisons between the findings with sUCr/SGD and sUCr/Osm

In the previous study utilizing NHANES 2011–2012[3], sUCr was 127.1 ± 84.0 mg/dL; UOsm, 649 ± 266 mOsm/Kg; and sUCr/Osm, 0.19 ± 0.08. In the current study using NHANES 2007–2008, sUCr was 123.6 ± 75.4 mg/dL; SGD, 16.8 ± 6.9; and sUCr/SGD, 7.0 ± 2.4. When sUCr levels in both studies are comparable, the ratio of UOsm to SDG is 38.6 (649/16.8) and the inverse ratio of sUCr/Osm to sUCr/SGD is 36.8 (7.0/0.19).

In the previous study[3], the mean value of the index of sUCr/Osm to esUCr/Osm was 1.05 ± 0.39; 4.7% of participants had their sUCr/Osm <0.08; 4.7% of participants had the sUCr/Osm indexed to individualized esUCr/Osm <0.50; and 3.3% had sUCr/Osm were simultaneously lower than both the lower reference limits of the above two left-sided reference intervals. Regarding the current study, the mean value of the sUCr/SGD indexed to esUCr/SGD was 1.03 ± 0.30; 4.7% had their sUCr/SGD <3.60; 4.6% had the sUCr/SGD indexed to individualized esUCr/SGD <0.60; and in 3.2%, sUCr/SGD was below the lower reference limits of both of the above left-sided reference intervals. Both studies showed similar distribution patterns and lower reference limits for the relative index values based on comparable data on age, sex ratio, BW, and biochemical values.

## Discussion

Analysis of the NHANES 2007–2008 dataset revealed consistent distribution patterns and lower limits for both sUCr/SGD and sUCr/Osm in the general adult population[3]. This consistency supports the potential of sUCr/SGD as a practical and accessible clinical tool. The exchange ratio of UOsm to SGD (36.8–38.6) aligns with the conventional assumption that a 35–40 mOsm/kg increase in UOsm corresponds to a 0.001 increase in USG^[13,14]^.

Multivariable regression analysis identified BW, Black race, and SCr as major positively correlates of sUCr/SGD, while age and BUN were major negatively correlated (Table 4). These findings reflect the physiological basis that urinary Cr excretion is influenced by muscle mass and renal function. However, discrepancies in the BUN and SCr correlations between univariable and multivariable models (Tables 3 and 4) likely stem from statistical confounding and multicollinearity – phenomena well-documented in studies examining renal biomarkers[20], consistent with findings from the previous study on sUCr and UOsm[3].

The rationale for using sUCr/Osm to estimate real-time urinary Cr excretion is based on three assumptions^[3,4]^: (1) daily urinary Cr excretion can be estimated using equations incorporating age, sex, race, and BW[6]; (2) individuals consuming a typical Western diet excrete 600–900 mOsm daily[7], or 10 mOsm/Kg[8]; and (3) urinary Cr and osmotic excretion rates exhibit synchronized circadian variations without postprandial peaks[9]. Therefore, in non-oliguric individuals with adequate urine output, sUCr/Osm could serve as a surrogate for the urinary Cr excretion rate.

USG is a routine urinalysis component and is more readily available than UOsm. Additionally, sUCr is often measured alongside proteinuria or albuminuria assessments. Until sUCr/Osm gains broader clinical acceptance, sUCr/SGD offers a more accessible alternative for estimating urinary Cr excretion.

The suitability of USG (SGD) as a substitute for UOsm has been widely debated^[10,11,14]^. By definition, UOsm quantifies of water-soluble particles per unit of water fraction in urine, whereas USG reflects urine density relative to pure water, determined by the solute numbers and molecular weight. The correlation between UOsm and USG depends on solute properties. Since each increase of 35–40 mOsm/kg in UOsm corresponds to a 0.001 rise in USG^[13,14]^, this relationship can be conceptually interpreted as an increase of 35–40 mmol of solutes per liter of urine equating to one gram of additional weight.

UOsm is approximately equal to twice the sum of the positively charged plus uncharged urinary solutes[21]. When Cl^-^ is the major anion in urine, other contributors include sulfate, phosphate, and some organic anions[22]. The average contributions of urinary solutes to Uosm are: NH_4_^+^ (6%), Na^+^ (19%), K^+^ (7%), Cl^-^ (21%), other anions (17%), and urea (25%)[23]. Given their molecular weights – Na^+^ (23), K^+^ (39), Cl^-^ (35.5), sulfate (96), phosphate (95), and urea (60) – SGD may underestimate UOsm in cases of high sodium excretion. Conversely, SGD may overestimate UOsm when urine contains substantial protein or glucose, as these heavy molecules disproportionately elevate USG readings[14].

Two methods commonly determine USG: refractometry and reagent strips. USG with a minimal reading to 0.001 in the present study was measured by refractometry, which measures light refraction proportional to solute concentration. Reagent strips, though convenient, have limitations. They measure USG in increments of 0.005, rely on ionic strength assumptions, and are affected by urine pH, leading to falsely high readings in acidic urine and vice versa[23].

Both sUCr/Osm and sUCr/SGD correlate positively with BW, Black race, and SCr, and negatively with age and BUN[3] (Table 4). Consequently, the esUCr/SGD equation also incorporates only significant covariables such as age, BW, Black race, BUN, and SCr. Given recent scrutiny of race-based medicine[24], race-neutral eGFR (estimated glomerular filtration rate) calculations have been adopted for kidney transplant eligibility since July 2022^[25,26]^. Using the same procedures and covariable selection criteria described previously, we derived a race-neutral esUCr/SGD formula: esUCr/SGD = 9.124 × (age, year)^−0.170^ × (BW, kg)^0.266^ × (BUN, mg/dL)^−0.290^ × (SCr, mg/dL)^0.635^. This formula yielded an adjusted R-squared of 0.287 (*P* < 0.001), with a mean esUCr/SGD of 6.7 ± 1.3 (range: 3.5–12.0, median: 6.6). The indexed sUCr/SGD was 1.04 ± 0.30 (range: 0.11–2.83, median: 1.02). Compared to the race-inclusive formula, the race-neutral version altered BUN and Cr exponents by approximately 10%, resulting in a 1.3% discrepancy in esUCr/SGD values. Of note, while the models offer practical estimation tools, the adjusted R-squared values (0.299 and 0.287) for race-inclusive and race-neutral esUCr/SGD formulas indicate modest predictive power, highlighting the need for further refinement in future studies.

Supplemental subgroup analysis examined sUCr/SGD variations by age, sex, and race (Table 5). Younger individuals (≤65 years), males, and Black participants exhibited higher lower reference limits for sUCr/SGD and its index, except that Black participants had a lower relative index using the race-inclusive formula. Within each subgroup, the ratios of the difference in the lower reference limit of the sUCr/SGD indexes are less than the ratio of the absolute value, consistent with normalization effects.

**Table 5:** Lower reference limits of the left-sided 95% reference intervals of the absolute value and relative index values of spot urine creatinine-to-specific gravity difference ratio, based on age, sex, and race

		Relative index value	
	Absolute value	Race-inclusive	Race-neutral
Age ≤ 65 yr vs. age > 65 yr, *n* = 2737 vs. 551	3.73 vs. 3.12	0.62 vs. 0.58	0.62 vs. 0.57
Male vs. female, *n* = 1776 vs. 1512	4.33 vs. 3.20	0.63 vs. 0.58	0.63 vs. 0.58
Black vs. non-Black, *n* = 587 vs. 2701	4.63 vs. 3.54	0.59 vs. 0.62	0.67 vs. 0.60

Several limitations of the current study should be noted. First, the original NHANES 2007–2008 dataset did not include concurrent UOsm measurements, which prevented direct comparisons between USG (expressed as SGD) and UOsm. This limitation hinders the confirmation of the correlation between sUCr/SGD and sUCr/Osm, a critical step in validating that the alternative method reflects urinary Cr excretion. Therefore, further head-to-head studies are needed to conclusively validate these correlations. Second, information regarding the conditions of the urine samples for glucosuria, pyuria, and hematuria was unavailable in the original NHANES 2007–2008 dataset. To minimize the potential impact of substantial protein or glucose, which possess molecular weights that exceed the expected range in typical urine samples, on the correlation between SGD and UOsm, the final dataset for this secondary analysis excluded those individuals with high urine albumin to Cr ratios or blood glucose levels. Third, the reference intervals or lower reference limits of sUCr/SGD would not be applicable to individuals who meet the exclusion criteria for anthropometric and laboratory parameters outlined in the Methods section. Fourth, the lack of relevant data precludes discounting the possibility of extreme dietary habits among subjects. Diets high in salt and protein raise solute excretion and osmotic load, while lower solute intake reduces them. Future studies should integrate validated dietary assessments (e.g. food frequency questionnaires or dietary recalls) to account for dietary variability and further validate sUCr/SGD as a marker for urinary Cr excretion. While hydration status has a negligible collective effect on the sUCr/SGD ratio due to the simultaneous and equivalent effects of urine dilution and concentration on sUCr and UOsm (SGD), extraordinary dietary osmotic load can significantly lower sUCr/SGD, resulting in an absolute value <3.6 or a relative index <0.60 due to increased urinary osmotic excretion.

In conclusion, the current study offers the reference intervals for sUCr/SGD in ordinary adults without substantial proteinuria or glucosuria. Therefore, sUCr/SGD can be proposed as a promising, cost-effective alternative to sUCr/Osm for estimating urinary Cr excretion rates in daily practice, particularly when a urine Cr value is accompanied with a USG measurement incidentally available rather than UOsm. A concise comparison of the features of sUCr/Osm and sUCr/SGD is summarized in Table 6. Given the practical implications for real-world settings, especially for primary care or low-resource contexts where USG is more feasible than UOsm, this cross-sectional study has preliminarily explored the features and distributions of sUCr/SGD and compared them to those findings in a closely relevant study on sUCr/Osm^3^, further head-to-head evaluations are warranted to validate the applications of these two urine parameters in various clinical scenarios.

**Table 6:** Comparisons between the features of spot urine creatinine-to-osmolality ratios (sUCr/Osm) and creatinine-to-specific gravity difference ratios (sUCr/SGD)

	Absolute value		Relative index value	
	sUCr/Osm	sUCr/SGD	sUCr/Osm	sUCr/SGD
1) Origin of dataset				
	NHANES 2011–2012, *n* = 3316	NHANES 2007–2008, *n* = 3288	NHANES 2011–2012, *n* = 3316	NHANES 2007–2008, *n* = 3288
2) Formula				
	Urine Cr divided by Osm	Urine Cr divided by SGD	sUCr/Osm divided by estimated sUCr/Osm^a^	sUCr/SGD divided by estimated sUCr/SGD^b^
3) Clinical application				
Tests needed	sUCr, UOsm	sUCr, USG	sUCr, UOsm	sUCr, USG
Cost	~USD $ 4.0	~USD $ 2.0	~USD $ 4.0	~USD $ 2.0
Data needed	Nil		Age, BW, BUN, SCr, Black race^c^	
Simplicity	Ready to calculate		Calculator needed	
Limitations	Extraordinary weight or intake	Extraordinary weight or intake Substantial urine protein or glucose	Extraordinary weight or intake	Extraordinary weight or intake Substantial urine protein or glucose
4) Physiological meaning				
	Excretion of Cr accompanied with per unit of osmole in urine	Excretion of Cr relative to per unit of SGD in spot urine	Normalization of sUCr/Osm considering personal features	Normalization of sUCr/SGD considering personal features
5) Statistical values				
Mean ± SD	0.19 ± 0.08	7.0 ± 2.4	1.05 ± 0.39^d^	1.03 ± 0.30^d^
			1.07 ± 0.41^e^	1.04 ± 0.30^e^
Median, IQR	0.18, 0.13–0.23	6.7, 5.3–8.3	1.02, 0.80–1.25^d^	1.00, 0.83–1.20^d^
			1.03, 0.81–1.26^e^	1.02, 0.83–1.21^e^
Range	0.02–0.78	1.1–19.8	0.14–4.75^d^	0.10–2.86^d^
			0.14–4.73^e^	0.11–2.83^e^
95% lower reference limit	0.08	3.6	0.50^d^	0.60^d^
			0.50^e^	0.60^e^

BUN, blood urea nitrogen; BW, body weight; Cr, creatinine; IQR, interquartile range; NHANES, National Health and Nutrition Examination Survey; SCr, serum creatinine; SD, standard deviation; SGD, specific gravity difference (the last two digits of the urine specific gravity); sUCr, spot urine creatinine; UOsm, urine osmolality; USG, urine specific gravity.

^a^ The value of sUCr/Osm estimated from relevant composite factors for an individual.

^b^ The value of sUCr/SGD estimated from relevant composite factors for an individual.

^c^ Black race is considered in the race-inclusive formula but ignored in the race-neutral formula.

^d^ Values using the race-inclusive formula.

^e^ Values using the race-neutral formula.

## Data Availability

The National Health and Nutrition Examination Survey, 2007–2008 (NHANES 2007–2008) contains data for 10 149 individuals of all ages. The files of the data have been available publicly on https://wwwn.cdc.gov/Nchs/Nhanes since September 2009.
